# FHY3 and FAR1 Act Downstream of Light Stable Phytochromes

**DOI:** 10.3389/fpls.2016.00175

**Published:** 2016-02-22

**Authors:** Hamad Siddiqui, Safina Khan, Bruce M. Rhodes, Paul F. Devlin

**Affiliations:** School of Biological Sciences, Royal Holloway University of LondonEgham, UK

**Keywords:** light, phytochrome, *Arabidopsis*, signal transduction, transcription

## Abstract

FHY3 and FAR1 are positively acting transcription factors that directly regulate expression of a number of target genes in *Arabidopsis thaliana*. Here, we looked at the regulation of one specific target gene, *ELF4*. We demonstrate that the action of FHY3 and FAR1 in upregulation of *ELF4* is light dependent. Furthermore, although FHY3 and FAR1 have been exclusively characterized as components of the phytochrome A signaling pathway because of their importance in regulating expression of phyA nuclear importers, we show that, as transcription factors in their own right, FHY3 and FAR1 act downstream of light stable phytochromes, phyB, phyD, and phyE. We demonstrate that light stable phytochrome acts in a red/far-red reversible manner to regulate the level of FHY3 protein. We also observed that *ELF4* shows specific FHY3 and FAR1-mediated light induction in the evening and we show that regulation by light stable phytochromes at this time is important as it allows the plant to maintain normal *ELF4* expression beyond dusk when the day length shortens, something which would not be possible through light labile phytochrome action. Without FHY3 and FAR1, *ELF4* expression falls rapidly at dusk and in short days this results in an early drop in *ELF4* expression, accompanied by a de-repression of an ELF4 target gene later in the night. Our results, therefore, demonstrate an important role for FHY3 and FAR1 as mediators of light stable phytochrome signaling.

## Introduction

Responses to light are one of the most important aspects of a plant’s adaptation to its environment. Germination, establishment, and the growth patterns throughout the life history of a plant are optimized to ensure the best possible acquisition of light energy for photosynthesis. Plants possess a wide range of photoreceptors in order to gather information about the light environment but among the most important are the red light and far red light-absorbing phytochromes.

Phytochromes are dimeric proteins, each phytochrome monomer binding a linear tetrapyrrole chromophore (Rockwell et al., 2006). Phytochrome exists in two photo-interconvertible forms, a red light-absorbing Pr form and a far red light-absorbing Pfr form. Conversion to the Pfr form results in nuclear entry and activation of target gene expression (Klose et al., 2015). Five phytochromes exist in *Arabidopsis thaliana* (*Arabidopsis*), named phytochrome A to phytochrome E (phyA to phyE; Franklin and Quail, 2010). PhyB–phyE are responsible for classical red/far red-reversible phytochrome responses, activated by red and deactivated by far red irradiation. PhyB–phyE Pfr is relatively stable (Sharrock and Clack, 2002) and, indeed, phytochrome B Pfr has been shown to remain active in darkness for up to 12 h following illumination (Hennig et al., 1999). PhyA, in contrast, shows activity in both red and far red and even in blue as Pfr levels sufficient to trigger phyA responses can be formed in all of these wavelengths (Whitelam et al., 1993; Casal et al., 1998). However, phyA is light labile and is rapidly degraded in the Pfr form. Thus, on cessation of illumination, phyA action ceases (Casal et al., 1998). None-the-less, despite this light lability, a significant pool of phyA is maintained in established seedlings where it continues to function throughout the life of the plant (Franklin et al., 2007).

Far-red elongated hypocotyl 3 (FHY3) and far-red-impaired response (FAR1) were originally identified as components of the phytochrome A (phyA) signal transduction pathway. Mutants in FHY3 and FAR1 show impaired inhibition of hypocotyl elongation in far red light (Whitelam et al., 1993; Hudson et al., 1999). The FHY3 and FAR proteins are close homologs and are part of a wider family of proteins showing similarity to mutator-like transposases (Hudson et al., 2003; Lin and Wang, 2004). The two proteins dimerize and function as transcription factors by binding to the FHY3/FAR1 binding sequence (fbs) to positively regulate expression of a number of genes (Wang and Deng, 2002; Lin et al., 2007; Ouyang et al., 2011). Indeed, the role of FHY3 and FAR1 in phyA signaling has been revealed to be due to their positive regulation of expression of the phyA nuclear importers, FHY1 and FHL (Lin et al., 2007). FHY3 and FAR1 act permissively in this respect: loss of either FHY3 or FAR1 results in a loss of target gene expression (Hudson et al., 2003; Li et al., 2011).

Among the FHY3 and FAR1 targets is the central clock gene, *ELF4*. The *fhy3*, *far1*, and *fhy3 far1* mutants show dramatically reduced, essentially arrhythmic expression of *ELF4* in constant light (Li et al., 2011). FHY3 and FAR1 are, therefore, proposed to be part of the light input pathway to the circadian clock acting downstream of phytochrome. However, although the role of FHY3 and FAR1 in positively regulating *ELF4* has been demonstrated, neither a light- nor phytochrome-specific role has yet been proven. This is particularly pertinent as FHY3 has been shown to be involved in a wide range of processes, not necessarily all light or phytochrome dependent (Huang et al., 2012; Wang and Wang, 2015; Wang et al., 2016).

We sought to examine the light dependency of FHY3 and FAR1 in the regulation of *ELF4* expression, originally aiming to confirm their importance in light input to the circadian clock. We demonstrated that FHY3 and FAR1, indeed, show a light dependent regulation of *ELF4* expression. Significantly, in this respect, we observed a previously unknown mode of FHY3 and FAR1 action downstream of light stable phytochromes, specifically, phyB, phyD, and phyE. Light stable phytochrome acts in a red/far-red reversible manner to regulate FHY3 protein level. We showed that *ELF4* expression is light responsive in the evening and demonstrated an important role for light stable phytochrome at this time in maintaining *ELF4* expression beyond dusk when day length is shortened. Without FHY3 and FAR1 action downstream of light stable phytochromes at this time, *ELF4* expression drops sharply at dusk. Consistent with this, we observed reduced repression of the ELF4 target gene, *PIF4*, later in the night in short days in *fhy3 far1* mutants. We, therefore, demonstrate that FHY3 and FAR1 are components of light stable phytochrome signaling and propose an argument that this may even be their primary mechanism of action in phytochrome signaling.

## Materials and Methods

### Plant Materials and Growth Conditions

The *fhy3-4 far1-2* double mutant of *Arabidopsis* is in the No-0 ecotype (Wang and Deng, 2002). Luciferase reporter lines containing *ELF4::LUC*, *mFBS ELF4::LUC*, and *FHY3::FHY3-LUC*/*fhy3-4* have been described previously (Li et al., 2011). Phytochrome mutant lines study were *phyB-1* (Koornneef et al., 1980), *phyB-1 phyD-1* (Devlin et al., 1999), *phyB-1 phyE-1* (Devlin et al., 1998).

In all experiments, seeds were sterilized in 30% bleach, 0.02% Triton X-100, sown on Murashige and Skoog (MS) medium containing 2% sucrose, then stratified for 3 days in darkness at 4°C before germination. All experiments were carried out at 21°C.

For RT-qPCR analysis of *ELF4* response to monochromatic red light, following stratification, seeds were germinated and grown in 12 h white light/12 h dark cycles for 7 days (equally mixed red and blue light, 100 μmol m^–2^ s^–1^). The plates were then transferred to red light at the fluence rates indicated for 4 days. All light used in this assay was provided by red (λ-max 660 nm) and blue (λ-max 450 nm) LEDs within Fytoscope FS 80-RGBIR Mini cabinets (Photon Systems International, Brno, Czech Republic).

For analysis of luciferase bioluminescence and for RT-qPCR analysis of *PIF4* expression, plants were germinated and grown in 12 h white light/12 h dark cycles for 7 days prior to treatment conditions. White light for this and for the treatment during RT-qPCR analysis of *PIF4* expression consisted of equally mixed red and blue light, 100 μmol m^–2^ s^–1^, provided by LEDs within Fytoscope FS 80-RGBIR Mini cabinets as above.

Light conditions during luciferase bioluminescence imaging experiments were provided within the imaging chamber by a custom-made LED rig providing red light (λ-max 660 nm, 40 μmol m^–2^ s^–1^), blue light (λ-max 450 nm, 40 μmol m^–2^ s^–1^) or white light consisting of equally mixed red and blue light. Timing of LED illumination within the chamber was controlled by a MLU2 digital timer (RS components, UK). However, EODFR treatment during imaging was carried out manually as described in Wang et al. (2011) using the same light sources. Far red irradiance was 15 μmol m^–2^ s^–1^. Seedlings were transferred to and from EODFR treatment under green safelight.

All light measurements were made using a StellarNet EPP2000-HR spectroradiometer.

### Luciferase Imaging

Luciferase imaging was carried out using a NightOwl ultra-cooled CCD (charge-coupled device) camera (Berthold Technologies, UK) as described by Wang et al. (2011) except that 1 day prior to commencement of imaging, seedlings were sprayed with a slightly higher concentration (5 mM) of d-luciferin dissolved in 0.01% Triton (1 ml per plate). Data were analyzed by using Winlight image analysis software version 2.17 (Berthold Technologies, UK). All data represent the findings of at least two independent experiments.

### RNA Extraction and Gene Expression Analysis

RNA extraction and qRT-PCR were carried out exactly as described previously (Wang et al., 2011). All gene expression values are expressed relative to either an *Arabidopsis UBQ10* or *IPP2* housekeeping control. All data represent the findings of at least two independent experiments. The following primers were used for qRT-PCR: *ELF4*, AGTTTCTCGTCGGGCTTTCACG and TAAGCTCTAGTTCCGGCAGCAC; *PIF4*, TCAGATGCAGCCGATGGAGATG and CGACGGTTGTTGACTTTGCTGTC; UBQ10, AAAGAGATAACAGGAACGGAAACATAGT and GGCCTTGTATAATCCCTGATGAATAAG; *IPP2*, TCGTGTTCCACGAGGACTCTAC and TCAACTGCACCTTCGATCTTAGC.

## Results

### The Action of FHY3 and FAR1 in Regulation of *ELF4* is Light Dependent and Specific to the Early Part of the Night

**FIGURE 1:**
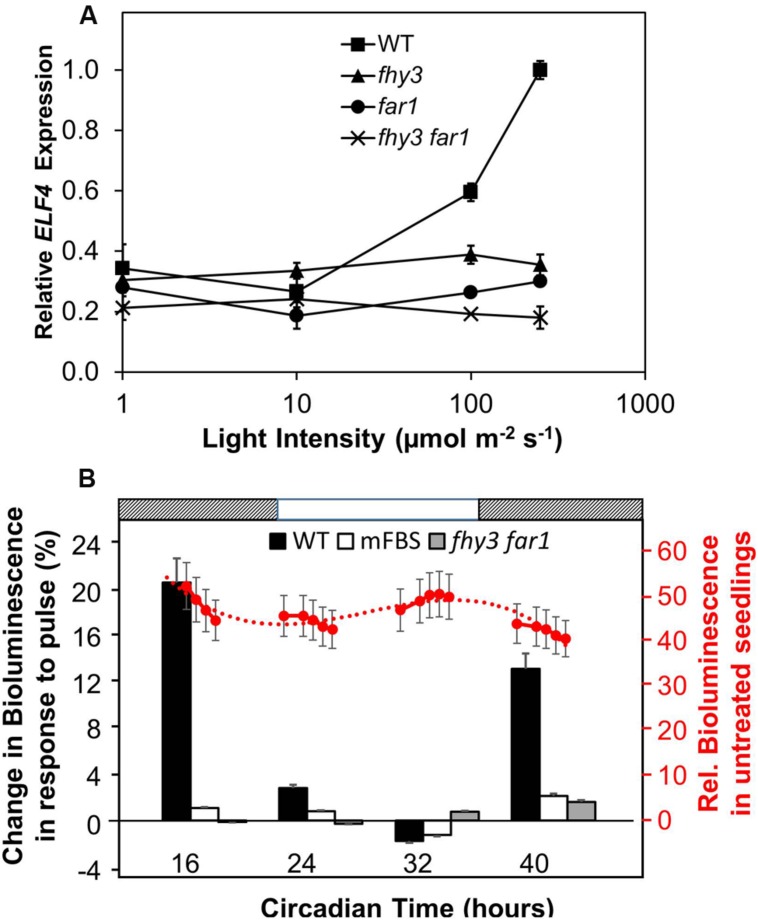
**(A)** The action of FHY3 and FAR1 in regulation of *ELF4* expression is light dependent. Wild-type (WT), *fhy3*, *far1*, and *fhy3 far1* mutant seedlings were grown in 12 h white light:12 h dark cycles for 1 week before transfer to constant red light for a 4 days at of a range of different intensities. Mean *ELF4* expression relative to *IPP2* measured by RT-qPCR for two independent replicates + standard error. **(B)** FHY3 and FAR1 directly regulate acute light responsive *ELF4* expression in the early part of the subjective night. WT and *fhy3 far1* seedlings, both expressing *ELF4::LUC*, as well as WT seedlings expressing an *ELF4::LUC* construct in which the FHY3/FAR1 binding site was mutated (mFBS), were grown in 12 h white light/12 h dark cycles for 1 week prior to transfer to constant darkness at dawn (Circadian Time 0). Seedlings were then given a 30 min pulse of 100 μmol m^–2^ s^–1^ red light at one of four time points over a circadian cycle. Left hand axis (black labels): percentage increase in bioluminescence recorded 30 min after the pulse at the four time points indicated. Right hand axis (red labels): mean relative bioluminescence of untreated WT seedlings, shown by red circles, and measured at a number of points over the time course to show the background pattern of *ELF4* expression. Dotted line represents trend line based on third order polynomial. Light and dark bars above the figure indicate the times of subjective day and night. *N* = at least 19 seedlings + standard error.

In order to carry out an initial investigation into the light dependency of FHY3 and FAR1 action in the positive regulation of *ELF4* expression in established seedlings, *fhy3*, *far1*, and *fhy3 far1* mutant seedlings were grown in 12 h white light:12 h dark cycles for 1 week before transfer to constant red for a further 4 days at of a range of different intensities. Wild-type (WT) seedlings showed an increase in *ELF4* transcript levels with increasing light intensity above 10 μmol m^–2^ s^–1^ (**Figure 1A**). In contrast, *ELF4* expression in *fhy3*, *far1*, and *fhy3 far1* mutant seedlings remained relatively unchanged across the range of light intensities. It is notable, though, that both the *fhy3* and *far1* monogenic mutants display a slightly greater *ELF4* expression than the *fhy3 far1* double mutant at light intensities above 10 μmol m^–2^ s^–1^ suggesting that some light responsiveness is retained in each of the single mutants. However, *ELF4* expression in *fhy3* or *far1* is still dramatically lower than that in WT at these intensities (**Figure 1A**), suggesting a synergistic action of both FHY3 and FAR1 is required for full responsiveness in WT. Most significantly, this confirms the light dependence of the action of FHY3 or FAR1 in regulating *ELF4* expression.

We then examined the response of WT and *fhy3 far1* mutant seedlings to red light pulses. As *ELF4* is an evening phased clock gene, we examined the effect of red pulses at various times over a 24 period in constant darkness. WT and *fhy3 far1* double mutant seedlings expressing the *ELF4::LUC* transgene were grown in 12 h light:12 h dark cycles for 1 week prior to transfer to constant darkness at dawn (Circadian Time 0, CT 0). Seedlings were then given a 30 min pulse of 100 μmol m^–2^ s^–1^ red light at one of four time points over a circadian cycle and induction of *ELF4::LUC* bioluminescence was recorded 30 min after the pulse. WT seedlings showed a strong induction of bioluminescence at CT 16 and 40, just after the subjective evening peaks of *ELF4* expression in constant darkness (**Figure 1B**). However, WT seedlings showed only minimal response at CT 24, corresponding to the minimum of *ELF4* expression at subjective dawn; and no response at CT 32, corresponding to the time at which *ELF4* expression is rising in the constant dark control. *fhy3 far1* seedlings by contrast showed little or no induction of *ELF4* expression at any of the time points tested (**Figure 1B**), demonstrating that FHY3 and FAR1 act downstream of red light photoreceptors in a time-of-day specific manner to promote expression of *ELF4*. In order to confirm the fact that FHY3 and FAR1 act in this way as a result of direct transcriptional regulation of *ELF4* expression via the fbs, we also examined responses in an *ELF4::LUC* construct in which the fbs sequences were mutated (*mFBS*; Li et al., 2011). The *mFBS* line also showed little or no induction of *ELF4* expression at any of the time points tested (**Figure 1B**). Thus, FHY3 and FAR1 act downstream of red light photoreceptors just after subjective dusk to upregulate *ELF4* expression via the fbs.

### FHY3 and FAR1 Buffer the Pattern of *ELF4* Expression Against Variation in Day Length

**FIGURE 2:**
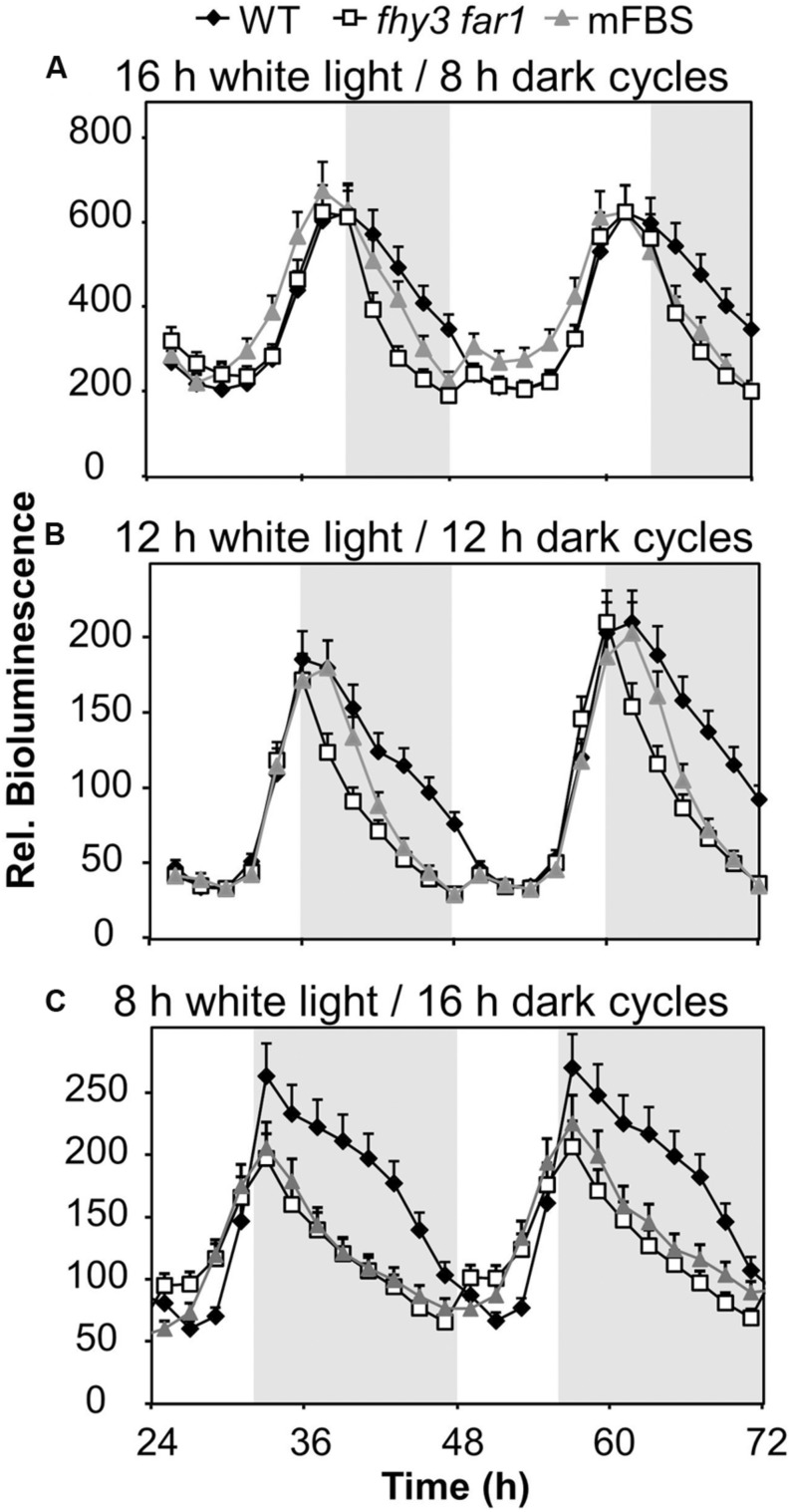
***ELF4* expression in *fhy3 far1* mutant seedlings is not buffered against changes in day length.** WT and *fhy3 far1* seedlings, both expressing *ELF4::LUC*, as well as WT seedlings expressing an *ELF4::LUC* construct in which the FHY3/FAR1 binding site was mutated (mFBS), were grown in 12 h white light/12 h dark cycles for 1 week before either **(A)** transfer to long days (16 h white light/8 h dark cycles); **(B)** maintenance in 12 h white light/12 h dark cycles; or **(C)** transfer to short days (8 h white light/16 h dark cycles). Values shown represent mean bioluminescence normalized to WT values at time zero of at least 17 seedlings + standard error.

We proposed that a strong positive regulation of the evening-phased gene, *ELF4*, by light dependent transcription factors, FHY3 and FAR1, around dusk would afford a mechanism by which the pattern of *ELF4* expression could adapt to day length as the time of dusk shifts with the seasons. As such, later dusk could be tracked courtesy of continued light input to the system which would maintain the *ELF4* peak for a longer duration and, therefore, allow the circadian clock to adapt to different day lengths. We therefore examined the pattern of expression from an *ELF4::LUC* construct in a range of day lengths in WT and in double mutant seedlings lacking FHY3 and FAR1. As expected, the peak of *ELF4* expression in WT seedlings closely followed the time of dusk (**Figure 2**). However, contrary to our predictions, the *ELF4* peak also faithfully tracked dusk in the *fhy3 far1* mutants (**Figure 2**) demonstrating that FHY3 and FAR1 were not involved in this aspect of light input to the clock. Significantly, though, while the WT peak remained roughly sinusoidal in all day lengths, we observed that the peak of *ELF4* expression in the double mutant showed a shark’s tooth expression pattern with a sharp drop immediately at dusk which becomes increasingly evident in shorter day lengths (**Figure 2**). Thus, rather than allowing the tracking of dusk, FHY3 and FAR1 appear to buffer the pattern of *ELF4* expression just following dusk against variation in day length.

As previously, in order to confirm that the effects of FHY3 and FAR1 here were mediated as a result of direct transcriptional regulation of *ELF4* expression, we also examined the response pattern of the (*mFBS* line). The *mFBS* line behaved in the same way as the *fhy3 far1* double mutants line in that *ELF4* expression dropped sharply following dusk, confirming that FHY3 and FAR1 act directly on the *ELF4* promoter in this buffering mechanism (**Figure 2**).

### The Role of FHY3 and FAR1 in Buffering the Pattern of *ELF4* Expression is Red Light Dependent and is Downstream of Light Stable Phytochrome Pfr

**FIGURE 3:**
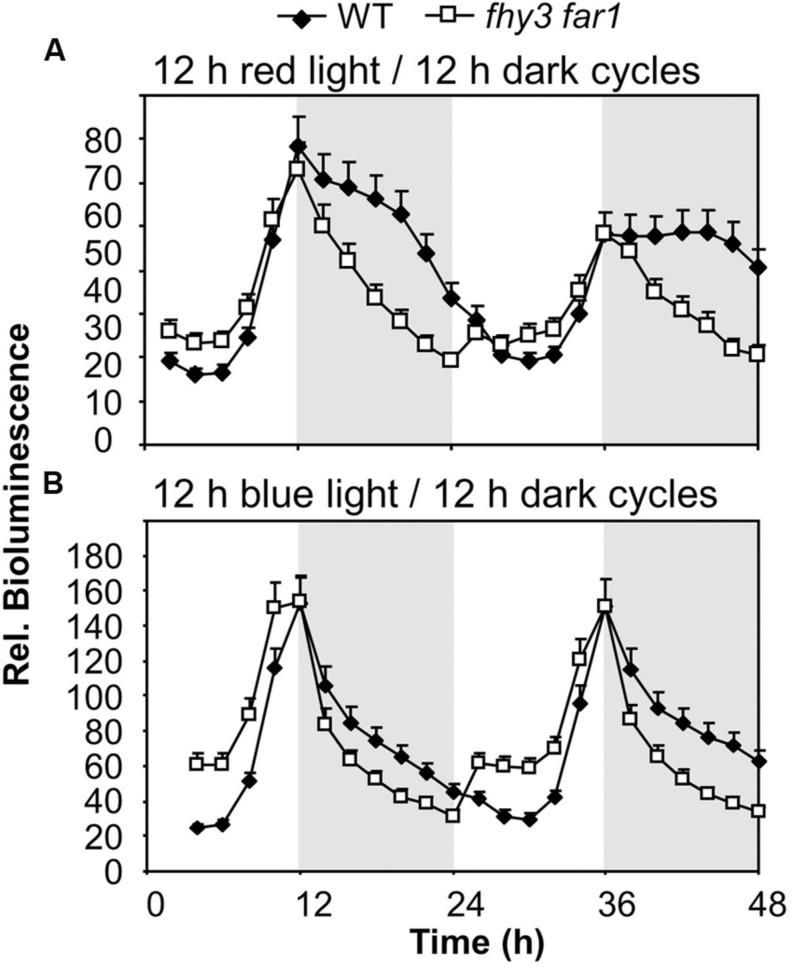
**Maintenance of the *ELF4* expression peak beyond dusk in WT seedlings is observed following red light but not blue light.** WT and *fhy3 far1* seedlings, both expressing *ELF4::LUC* were grown in 12 h white light/12 h dark cycles for 1 week before transfer to either **(A)** 12 h red light/12 h dark cycles; or **(B)** 12 h blue light/12 h dark cycles. Values shown represent mean bioluminescence normalized to WT values at time zero of at least 19 seedlings + standard error.

To determine whether the role of FHY3 and FAR1 in buffering the pattern of *ELF4* expression was red light dependent we examined whether the phenomenon was observed in both red/dark cycles and blue/dark cycles. Under 12 h red/12 h dark cycles we observed the same sinusoidal shape to the *ELF4* expression peak in WT seedlings as was observed in white light/dark cycles, with a peak of expression at dusk. The *fhy3 far1* double mutant seedlings also displayed the same pattern of *ELF4* expression as they had shown in white light/dark cycles, with a sharp drop in *ELF4* expression immediately at dusk (**Figure 3A**). However, under 12 h blue/12 h dark cycles, both WT and *fhy3 far1* double mutant seedlings showed a sharp drop in *ELF4* expression immediately at dusk. In blue/dark cycles the WT exactly phenocopied the *fhy3 far1* double mutant suggesting that the buffering of the *ELF4* peak by FHY3 and FAR1 is dependent on red light (**Figure 3B**).

**FIGURE 4:**
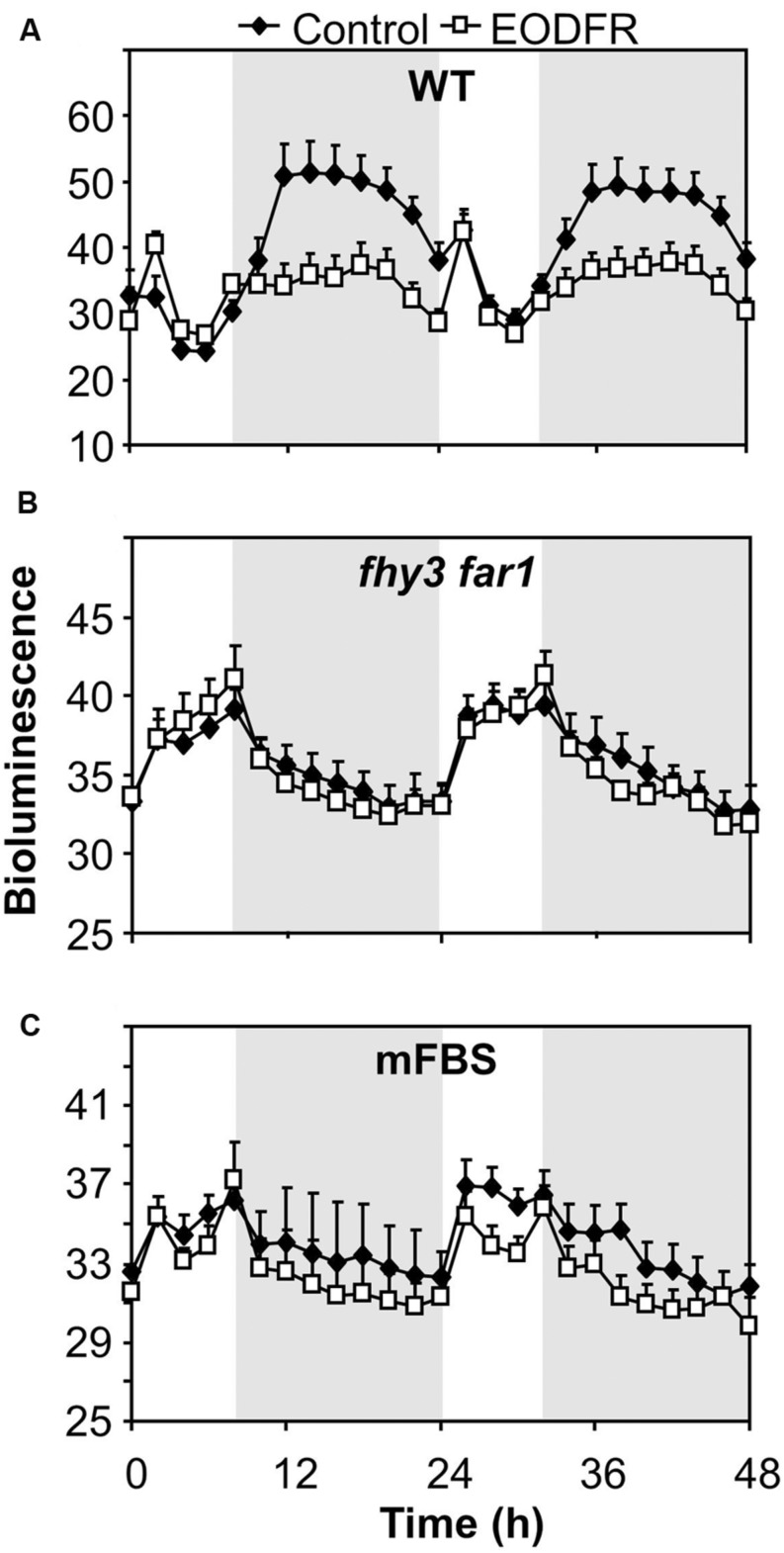
**Maintenance of the *ELF4* expression peak beyond dusk in WT seedlings is red/far red reversible.** **(A)** WT seedlings expressing *ELF4::LUC*, **(B)** *fhy3 far1* seedlings expressing *ELF4::LUC*, and **(C)** WT seedlings expressing an *ELF4::LUC* construct in which the FHY3/FAR1 binding site was mutated (mFBS), were grown in 12 h white light/12 h dark cycles for 1 week before transfer to short days (8 h white light/16 h dark cycles). Seedlings were either treated with a 15 min end of day far red pulse (EODFR) or maintained in short days without EODFR treatment (Control). Values shown represent mean relative bioluminescence of at least 15 seedlings + standard error.

The fact that the action of FHY3 and FAR1 on *ELF4* expression is dependent on red light suggests a role for phytochrome. However, FHY3 and FAR1 maintain the *ELF4* peak beyond dusk in WT seedlings meaning that, during the key period when FHY3 and FAR1 are required, light is not incident on the seedlings. Instead, the action of FHY3 and FAR1 appears to *follow* a period of red light. Such a phenomenon more-specifically suggests the action of stable phytochrome Pfr, implicating phyB, C, D, or E. In order to test the possible role of FHY3 and FAR1 in light-stable phyB-E signaling, an End of Day Far red light (EODFR) experiment was performed. An EODFR pulse will have the effect of severely depleting the pool of stable Pfr at dusk. *ELF4::LUC* expression was monitored in WT and *fhy3 far1* mutant plants grown in 8 h red light/16 h dark cycles with or without an EODFR pulse. WT seedlings treated with an EODFR pulse showed a loss of the sinusoidal pattern of the *ELF4* expression peak (**Figure 4A**), such as was seen previously in *fhy3 far1* double mutant seedlings (**Figure 2C**). Upon EODFR treatment, *ELF4* expression in WT seedlings stopped rising and plateaued at a much lower level through the subsequent night. Conversely, EODFR treatment had no effect on *fhy3 far1* double mutants or on *ELF4* expressed from the *mFBS* line, confirming a constitutive lack of stable Pfr signaling in these lines (**Figures 4B,C**).

**FIGURE 5:**
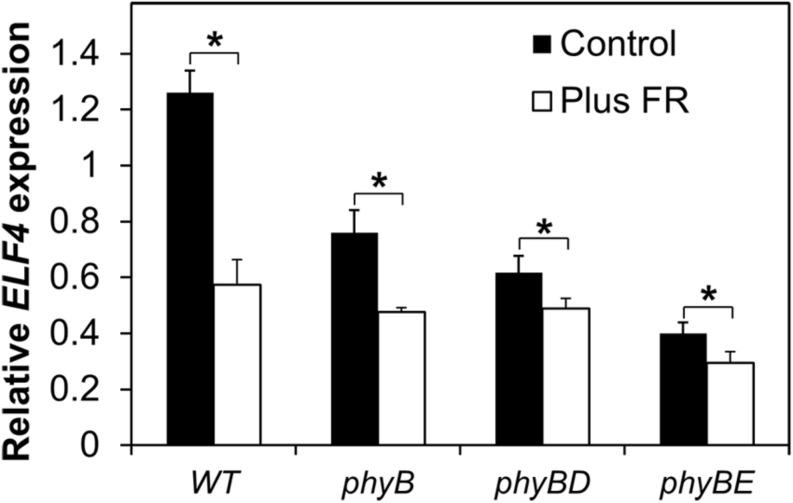
**Maintenance of the *ELF4* expression peak beyond dusk in WT seedlings involves phyB, phyD, and phyE.** Seedlings were grown in 12 h white light/12 h dark cycles for 1 week before transfer to short days (8 h white light/16 h dark cycles). Seedlings were either treated with a 15 min EODFR or maintained in short days without EODFR treatment (Control). Expression of *ELF4* in WT *phyB*, *phyB phyD*, and *phyB phyE* mutant seedlings 4 h following dusk. Values shown represent mean expression of *ELF4* relative to WT level at dusk, normalized to *IPP2* for minimum two replicates + standard error. (^∗^*p* < 0.05 using a heteroscedastic *t*-test).

In order to examine which phytochrome is responsible for regulation of *ELF4* expression following dusk, we examined a range of phytochrome deficient mutants. WT and phytochrome deficient mutants were grown in 8 h red light/16 h dark cycles with or without an EODFR treatment. Measurement of *ELF4* expression by qPCR at a single time-point 4 h after dusk was used to examine effectiveness of an EODFR pulse. As previously (**Figure 4**), WT seedlings showed a strong reduction in *ELF4* expression as a result of an EODFR pulse (**Figure 5**). Untreated *phyB* mutants showed lower *ELF4* expression than WT at this point and, crucially, *phyB* mutants showed a greatly reduced response to EODFR indicating the involvement of phyB in this response (**Figure 5**). None-the-less, phyB mutants still showed a significant EODFR response indicating the additional action of other light stable phytochromes. The *phyB phyD* double mutant seedlings showed a further drop in *ELF4* transcript compared to the *phyB* monogenic mutant and, significantly, showed a further reduced response to EODFR, implying some redundancy between phyB and phyD in the regulation of *ELF4* expression. The *phyB phyE* double mutant showed a yet more dramatic reduction in *ELF4* expression compared to *phyB* and also showed a reduced response to EODFR (**Figure 5**), together suggesting that phyB, phyD, and phyE all act following dusk to positively regulate *ELF4* expression, allowing the maintenance of a sinusoidal expression pattern in short days.

### Stable Phytochrome Regulates FHY3 Protein Levels

It was previously observed that FHY3 protein levels are regulated by light (Li et al., 2011). No light regulation of *FHY3* mRNA was observed by Li et al. (2011), demonstrating that this regulation occurs at the level of the protein itself. We, therefore, examined whether the action of phytochrome in triggering FHY3 activity in our assay could be mediated via a regulation of FHY3 protein levels. Seedlings containing an *FHY3::FHY3-LUC* construct, expressing an FHY3-LUC fusion protein under the control of the *FHY3* promoter (Li et al., 2011) in the background of the *fhy3* mutation were used in order to follow FHY3 protein levels.

**FIGURE 6:**
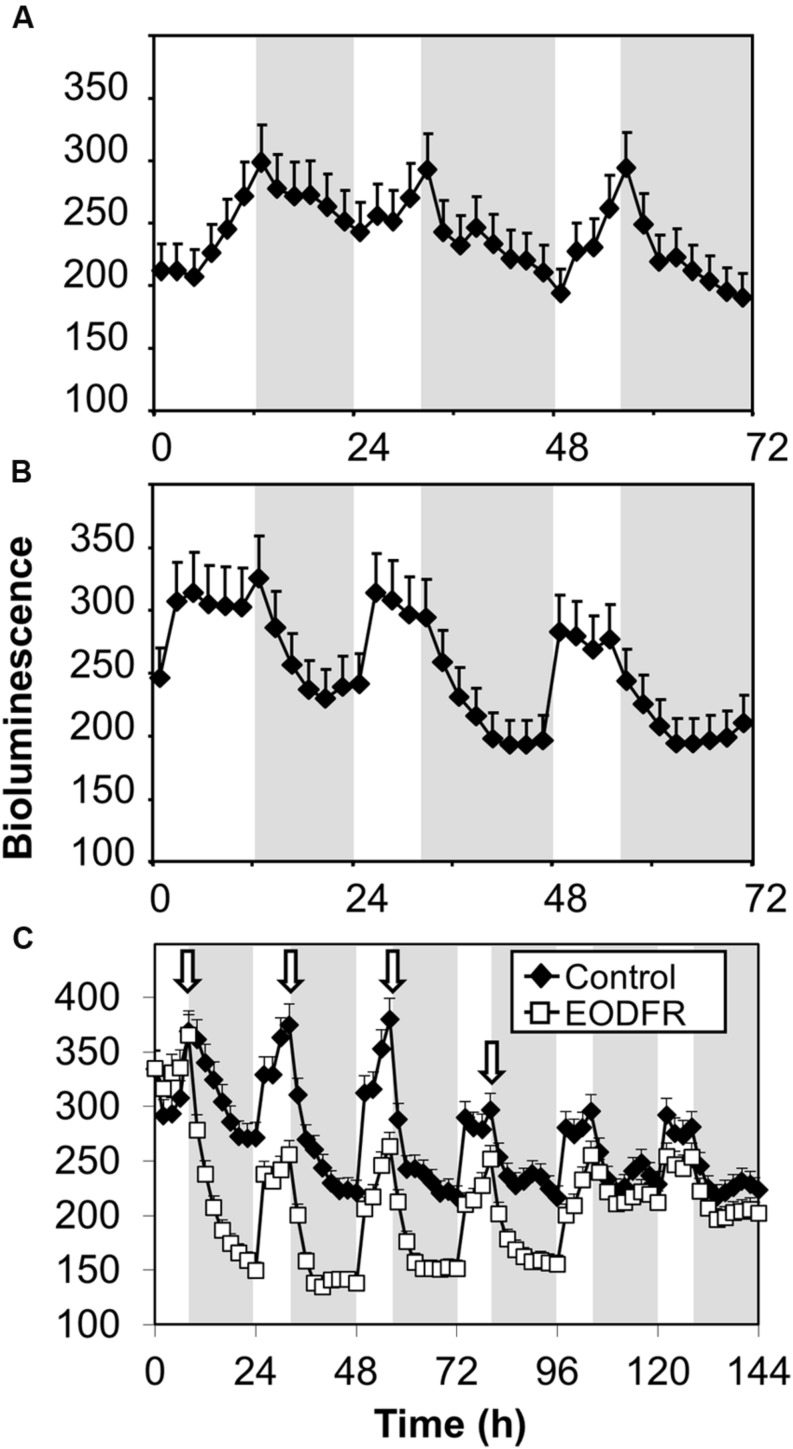
**Maintenance of FHY3 protein beyond dusk is red light-specific and shows red/far red reversibility.** Seedlings of the *fhy3* mutant expressing an *FHY3::FHY3-LUC* translational-fusion reporter construct were grown in 12 h white light/12 h dark cycles for 1 week before transfer to either **(A)** red light/dark cycles; **(B)** blue light/dark cycles; or **(C)** 8 h white light/16 h dark cycles with a 15 min EODFR or without (Control). Arrows represent times of EODFR treatment. Values shown represent mean relative bioluminescence of at least 20 seedlings + standard error.

*FHY3::FHY3-LUC* seedlings were grown in red/dark or blue/dark cycles. In red/dark cycles of 12 h red/12 h dark then 8 h red/16 h dark, bioluminescence from the FHY3-LUC fusion protein showed an evening-phased peak which gradually declined throughout the night (**Figure 6A**). In contrast, in blue/dark cycles of 12 h blue/12 h dark then 8 h blue/16 h dark, the evening peak of bioluminescence from the FHY3-LUC fusion protein dropped sharply at dusk reaching a basal level after just 6–8 h (**Figure 6B**). This pattern follows that of the FHY3 target gene *ELF4* in such conditions (**Figure 3**) and, therefore, strongly supports the proposal that regulation of FHY3 protein levels by light stable phytochrome Pfr is at least partly responsible for the red/far-red reversible regulation of FHY3 activity in our *ELF4* assay. It is notable, however, that a pronounced dawn acute induction of bioluminescence from the FHY3-LUC fusion protein was observed in blue/dark cycles, suggesting involvement of a blue light receptor during the day time, though, clearly not following dusk.

If *FHY3::FHY3-LUC* plants grown in white/dark cycles were treated with an EODFR pulse an immediate sharp drop in bioluminescence from the FHY3-LUC fusion protein followed (**Figure 6C**). After the first treatment with EODFR, levels fell and were not able to recover fully during the following day. Each subsequent EODFR treatment given over 4 days caused a similar immediate drop in bioluminescence from the FHY3-LUC fusion protein to a basal level. Following two further cycles of white/dark without EODFR treatment, bioluminescence from the FHY3-LUC fusion protein recovered to follow a WT pattern again (**Figure 6C**). Again, this pattern follows that of the FHY3 target gene *ELF4* in these conditions and suggests that light stable phytochrome regulation of FHY3 protein levels is at least partly responsible for the red/far-red reversible regulation of FHY3 activity.

### Growth of *fhy3 far1* Mutant Seedlings in Short Days Reveals Early Derepression of an ELF4 Target

**FIGURE 7:**
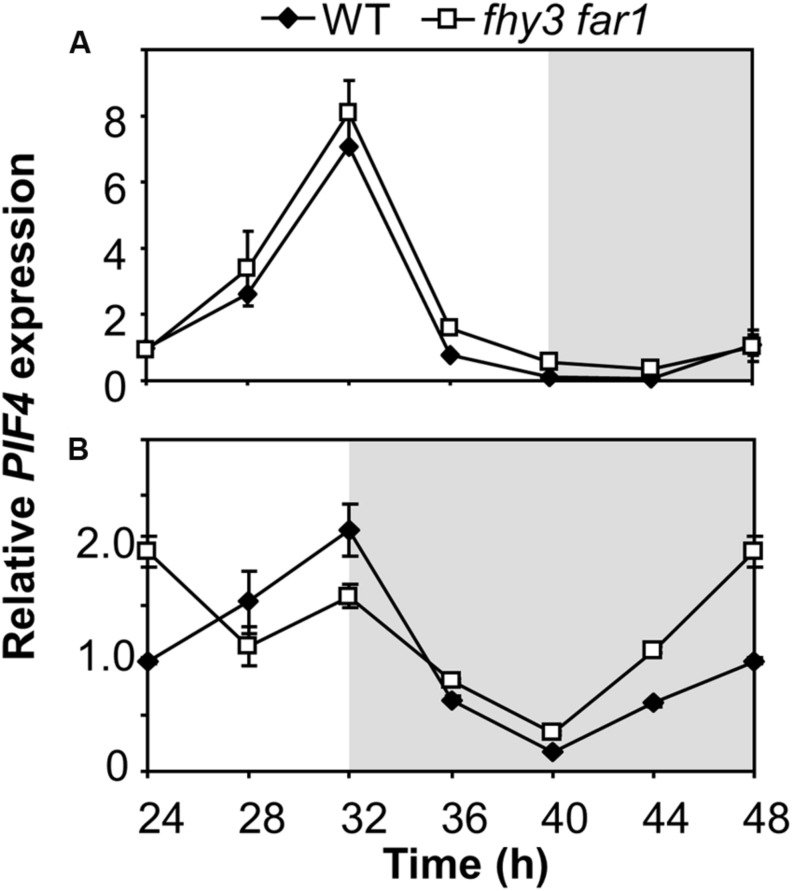
**Growth of *fhy3 far1* mutant seedlings in short days reveals an early release of *PIF4* repression.** Expression of *PIF4* relative to *UBQ10* as measured by RT-qPCR in WT and *fhy3 far1* seedlings grown in 12 h white light/12 h dark cycles for 1 week before transfer to either long days (16 h white light/8 h dark cycles; **A**); or short days (8 h white light/16 h dark cycles; **B**). Values shown represent mean expression normalized to *UBQ10* for three replicates ± standard error.

The mechanism of FHY3 and FAR1 action downstream of light stable phytochrome Pfr in buffering *ELF4* expression following dusk in short days would be expected to have a knock-on effect on any ELF4 target genes. ELF4 mediates direct night-time repression of expression of *PIF4* (Nusinow et al., 2011) and so we examined *PIF4* expression patterns in WT and *fhy3 far1* mutants in both longs and short days. We observed the expected cyclic pattern of *PIF4* expression in both long and short days, with a *PIF4* showing a peak of expression 8 h after dawn (**Figure 7**). *PIF4* levels were almost identical in both WT and *fhy3 far1* seedlings in long days (**Figure 7A**). However, we noted a significantly earlier rise in *PIF4* expression prior to dawn specifically in *fhy3 far1* seedlings grown in short days (**Figure 7B**) consistent with the observed early drop in *ELF4* expression seen at dusk in these conditions (**Figure 2C**).

## Discussion

The transcription factors, FHY3 and FAR1, have been shown to play an important role in the clock in positively regulating transcription of the evening-phased *ELF4* gene, an action that is required to maintain rhythmicity in constant light (Li et al., 2011). FHY3 and FAR1 have been shown to be key components of the phyA signaling pathway; hence, the assumption has been that they play a role in light input to the clock; however, this role had not yet been empirically proven. We set out to demonstrate the light dependency of FHY3 and FAR1 action in the upregulation of *ELF4* expression. Our findings demonstrate clear light-dependent action of FHY3 and FAR1 in the regulation of *ELF4*, with FHY3 and FAR1 acting permissively. We show that FHY3 and FAR1 act directly via the fbs in red light to upregulate *ELF4*, specifically in the early part of the subjective night. Loss of *ELF4* expression at this time has a knock on effect on downstream ELF4 target gene, *PIF4*. We demonstrate that this action of FHY3 and FAR1 is regulated in a red/far-red reversible manner by phyB, phyD and phyE. This can be at least in-part explained by our demonstration that FHY3 protein levels are also controlled in a red/far-red reversible manner. Most significantly, however, this represents the first demonstration of FHY3 and FAR1 as components of light stable phytochrome signaling pathways.

The permissive action of FHY3 and FAR1 in regulation of *ELF4* expression (**Figure 1A**) is consistent with previous observations of permissive action of these two transcription factors (Hudson et al., 2003; Li et al., 2011). However, *fhy3* or *far1* monogenic mutants did show slightly greater *ELF4* expression than the double mutant at light intensities above 10 μmol m^–2^ s^–1^. This is still dramatically lower than *ELF4* expression in WT seedlings at higher light intensities suggesting that any action of FHY3 alone or FAR1 alone is minimal in this response and that a synergistic or cooperative action between the two transcription factors is required for correct function.

As part of our investigation, we hypothesized that this light responsive action of FHY3 and FAR1 upregulating *ELF4* around dusk might form a means by which plants could track a later dusk in lengthening days during spring by delaying the peak of *ELF4* expression under these conditions. We had speculated that this might allow the circadian clock to adapt to different day lengths. Conversely, we found that plants lacking FHY3 and FAR1 responded normally in terms of tracking dusk meaning that FHY3 and FAR1 are not involved in this aspect of light regulation of *ELF4*. However, we observed that, whereas WT seedlings displayed a sinusoidal decline in *ELF4* expression following the dusk peak, the *fhy3 far1* double mutant showed a sharp drop in *ELF4* expression at dusk and this was particularly apparent under short day conditions. FHY3 and FAR1, therefore, act to buffer the *ELF4* peak following dusk to maintain *ELF4* expression into the early part of the night and this seems especially important in short days. In long days at dusk the expression of *ELF4* will be beginning to be suppressed by the accumulating circadian clock components, CCA1 and LHY (Li et al., 2011), meaning that levels will fall rapidly even in WT seedlings in spite of any positive effect of FHY3 and FAR1 action. Thus, little effect of FHY3 and FAR1 deficiency would be expected in long days. However, in short days, dusk is reached prior to the commencement of accumulation of *CCA1* and *LHY* meaning that, in WT, the positive effects of FHY3 and FAR1 would be expected to be much more apparent just after dusk.

End of day far red light treatment given to WT seedlings grown in short days was able to replicate the effect of FHY3 and FAR1 deficiency, in that WT seedlings treated with EODFR showed a loss of the sinusoidal pattern of the *ELF4* expression peak. It is notable, though, that *ELF4* expression in WT seedlings stopped rising and plateaued rather than dropping as it did in the *fhy3 far1* mutant. This is consistent with the fact that EODFR treatment does not remove all light stable phytochrome Pfr. A small amount of Pfr is known to remain in broadband far red since Pr and Pfr have overlapping absorption spectra below 730 nm (Smith and Holmes, 1977). It is likely that this a small amount of Pfr signaling contributes to the maintenance of some promotion of *ELF4* expression which would not be seen if the signaling pathway were knocked out completely due to mutation.

The action of FHY3 and FAR1 downstream of light stable phytochromes, phyB, phyD, and phyE raises the question of how light regulates the activity of these transcription factors. We have shown that FHY3 protein levels are regulated in a red/far-red reversible manner by the action of light stable phytochromes. There is no light or temporal regulation of *FHY3* transcript (Li et al., 2011); thus, our observation of light stable phytochrome regulation of bioluminescence from an FHY3-LUC fusion protein means that there is a level of regulation of the FHY3 protein itself. It is important to note that the reaction of luciferase fusion reporter with its substrate, luciferin, inactivates the luciferase enzyme. The measured bioluminescence is, therefore, recording previously unreacted luciferase only. However, this does not necessarily mean that we are simply measuring light regulation of translation. It is quite possible that this could reflect different rates of turnover too. Significantly, the luciferase-luciferin reaction is not instantaneous. This is evidenced by the “pre-spray effect” observed when plants are sprayed 24 h prior to commencement of an imaging experiment as is standard practice to inactivate the previously accumulated luciferase. If imaged immediately after this pre-spray, plants give off extremely high levels of bioluminescence. It commonly takes over 12 h for the pre-spray to inactivate all of the previously produced luciferase in 5-day-old seedlings (Somers et al., 1998). The delay in reaction of newly synthesized luciferase means that there would be sufficient time for degradation effects to also have an impact on bioluminescence. It is likely that a rapid turnover of a luciferase-linked protein would lead a much lower level of luciferase bioluminescence being recorded during a 25 min imaging window, just as reduced production would. One possibility is, therefore, that the FHY3 protein is stabilized by light stable phytochrome action. This could only be definitively concluded by future generation and analysis of a *35S::FHY3-LUC* line but this is a very common mechanism for the transmission of light signals, with a number of phytochrome signaling components being controlled in this way (Franklin and Quail, 2010). It has been demonstrated that there is a direct interaction between the FHY3 and FAR1 proteins and the phyA protein (Saijo et al., 2008) and so it would be important in the future to investigate whether other phytochromes also show such an interaction which might mediate this effect. Furthermore, it would be interesting to examine whether this might involve the COP1 protein which acts as an E3 ubiquitin ligase and directly targets a number of other light signaling transcription factors for degradation in darkness (Osterlund et al., 2000; Bauer et al., 2004; Yang et al., 2005). Indeed, both phyA and phyB have been shown to mediate the light-induced reduction of COP1 in *Arabidopsis* nuclei (Osterlund and Deng, 1998).

We also show that the sharp drop in *ELF4* expression at dusk in short days in *fhy3 far1* mutants precedes a subsequent aberrant repression of *PIF4* in the later part of the night. *PIF4*, a direct negative target of ELF4, shows an earlier rise prior to dawn in *fhy3 far1* mutants in short days. Higher *PIF4* expression levels in the later part of the night following the lower *ELF4* levels at the beginning of the night in *fhy3 far1* mutants in short days is consistent with the proven role of ELF4 as a repressor of *PIF4* expression during the subjective night (Nusinow et al., 2011).

**FIGURE 8:**
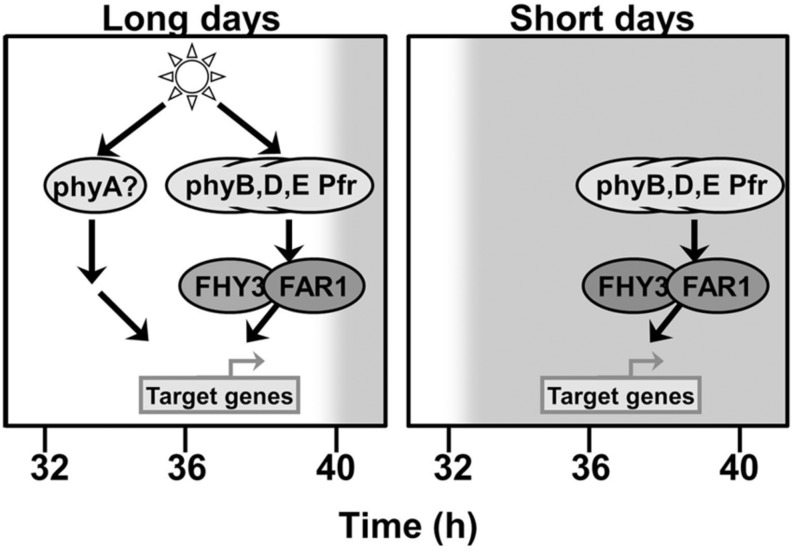
**FHY3 and FAR1 act downstream of phyB, phyD, and phyE Pfr to maintain *ELF4* expression into the early part of the night following a short day.** An additional pathway acts to compensate for the loss of FHY3 and FAR1 at this same time in long days. This additional pathway can be activated by either red or blue light but is triggered only in the continued presence of light, suggesting the involvement of phyA as a photoreceptor.

The fact that the deficiency in FHY3 and FAR1 is only observed to affect the pattern of expression of *ELF4* following dusk suggests that another photoreceptor signaling pathway which does not require FHY3 and FAR1 is able to maintain *ELF4* expression prior to this as long as light is incident on the plant. This maintenance of *ELF4* expression until dusk is also observed under both red light/dark cycles and blue light/dark cycles. The action of continuous red light is strongly suggestive of phyA signaling as phyA Pfr is rapidly degraded and so ceases to function when illumination stops (Casal et al., 1998). PhyA also shows strong activity under blue wavelengths (Whitelam et al., 1993), suggesting that it may be the photoreceptor responsible for upregulation of *ELF4* expression during illumination in both red and blue. Consistent with this, *ELF4* expression has been previously shown to be phyA regulated (Tepperman et al., 2001). **Figure 8** attempts to combine these proposals to a comprehensive scheme to explain the mechanisms of regulation of *ELF4* expression by phytochromes and the role of FHY3 and FAR1 in both long and short days. The proposed continued action of phyA in an *fhy3 far1* mutant does, of course, raise one additional issue, though. FHY3 and FAR1 are components of phyA signal transduction and, as a consequence, a number of phyA responses are severely impaired in the *fhy3* or *far1* monogenic mutants (Whitelam et al., 1993; Hudson et al., 1999). However, it has been shown that a number of phyA mediated physiological responses do not require FHY3 (Yanovsky et al., 2000). Similarly, the action of FHY3 and FAR1 in phyA signal transduction has been shown to be due to their action in promoting expression of *FHY1* and *FHL* which facilitate nuclear entry of phyA Pfr (Lin et al., 2007) but other factors have also been shown to be capable of facilitating nuclear entry of phyA Pfr. PIF1 and PIF3 have been shown to mediate nuclear entry of a phyA N-terminal fragment in a cell-free system (Pfeiffer et al., 2012) and, consistent with this, several other phyA dependent nuclear responses have been observed in an *fhy1 fhl* mutant (Kami et al., 2012; Klose et al., 2015).

Our demonstration here that FHY3 and FAR1 act as signaling components downstream of light stable phytochromes also raises some interesting points for the field of photomorphogenesis research. Our findings reveal that, by acquiring phytochrome responsivity, the evolution of FHY3 and FAR1 from transposases has involved two steps in a relatively short space of time. They have not simply acquired roles as constitutive activators of transcription needed for phyA function, but they have also acquired active roles as part of the signal transduction pathway itself downstream of light stable phytochromes. In addition, the findings raise a question as to whether this activity may mean that FHY3 and FAR1 have an important role in de-etiolation responses downstream of light stable phytochromes. We demonstrated a link between FHY3/FAR1 and PIF4, an important player in de-etiolation, while the potential role of COP1 in regulation of FHY3 protein stability also warrants further investigation, not least because of the important role of COP1 in de-etiolation. Such a role for COP1 would make its role even more complex and nuanced in the control of de-etiolation. Some effect of FHY3 and FAR1 on gene expression in darkness has actually been observed previously too. Hudson et al. (2003) showed altered gene expression in *fhy3* and *far1* mutants germinated in darkness. This raises the question as to whether this may be explained as light stable phytochrome Pfr in the seed activating FHY3 and FAR1 to trigger the activation of these gene targets. Light stable phytochrome Pfr is known to be contained even within dry seed and can have significant effects on germination. Furthermore, it is common to use a pulse of white light to synchronize germination 24 h prior to assays of phytochrome responses. It has been observed that such pre-treatment of seeds has a significant effect on subsequent gene expression responses (Leivar et al., 2008). It may be that this action involves activation of FHY3 and FAR1 as well as PIFs as demonstrated by those authors. Not least, such activation of FHY3 and FAR1 would be expected to trigger FHY1 and FHL production so as to allow phyA signaling. Consistent with this, treatment of seeds with a white light pulse 24 h prior to transfer to far red light, greatly enhances subsequent phyA signaling effects in promoting germination (Devlin et al., 1995).

## Conclusion

We show here that FHY3 and FAR1, originally identified as phyA signaling components, additionally act downstream of light stable phytochromes to promote *ELF4* expression in a light dependent manner. FHY3 and FAR1 are essential for phyB, phyD, and phyE Pfr action in maintenance of a normal expression pattern of *ELF4* following dusk in short days. However, one final whimsical thought occurs to the authors. Given the fact that the action of FHY3 and FAR1 in phyA signaling is due to their action as transcription factors to upregulate FHY1 and FHL, it could be said that this does not represent a direct role for FHY3 and FAR1 in phyA signaling itself, but, rather, an indirect role. In contrast, what we show here could be considered as the first evidence of their direct action as part of a light signaling pathway, in which case it might even be valid to reassign FHY3 and FAR1 as exclusively light stable phytochrome signaling components.

## Author Contributions

PD and HS contributed to project design. HS and SK carried out the bioluminescence analyses of the LUC reporter lines. HS, SK, and BR carried out the qRT-PCR assays. HS and PD wrote the manuscript. All authors discussed the results and commented on the manuscript.

## Conflict of Interest Statement

The authors declare that the research was conducted in the absence of any commercial or financial relationships that could be construed as a potential conflict of interest.
